# The RNA surveillance proteins UPF1, UPF2 and SMG6 affect HIV-1 reactivation at a post-transcriptional level

**DOI:** 10.1186/s12977-018-0425-2

**Published:** 2018-06-28

**Authors:** Shringar Rao, Raquel Amorim, Meijuan Niu, Abdelkrim Temzi, Andrew J. Mouland

**Affiliations:** 1 HIV-1 RNA Trafficking Laboratory, Lady Davis Institute at the Jewish General Hospital, Montreal, QC H3T 1E2 Canada; 2 Department of Microbiology and Immunology, McGill University, Montreal, QC H3A 2B4 Canada; 3 Department of Medicine, McGill University, Montreal, QC H3A 0G4 Canada

**Keywords:** HIV-1 latency, RNA surveillance proteins, HIV-1 genomic RNA stability, Post-transcriptional regulation, Nonsense-mediated mRNA decay, NMD, UPF1, UPF2, SMG6

## Abstract

**Background:**

The ability of human immunodeficiency virus type 1 (HIV-1) to form a stable viral reservoir is the major obstacle to an HIV-1 cure and post-transcriptional events contribute to the maintenance of viral latency. RNA surveillance proteins such as UPF1, UPF2 and SMG6 affect RNA stability and metabolism. In our previous work, we demonstrated that UPF1 stabilises HIV-1 genomic RNA (vRNA) and enhances its translatability in the cytoplasm. Thus, in this work we evaluated the influence of RNA surveillance proteins on vRNA expression and, as a consequence, viral reactivation in cells of the lymphoid lineage.

**Methods:**

Quantitative fluorescence in situ hybridisation—flow cytometry (FISH-flow), si/shRNA-mediated depletions and Western blotting were used to characterise the roles of RNA surveillance proteins on HIV-1 reactivation in a latently infected model T cell line and primary CD4+ T cells.

**Results:**

UPF1 was found to be a positive regulator of viral reactivation, with a depletion of UPF1 resulting in impaired vRNA expression and viral reactivation. UPF1 overexpression also modestly enhanced vRNA expression and its ATPase activity and N-terminal domain were necessary for this effect. UPF2 and SMG6 were found to negatively influence viral reactivation, both via an interaction with UPF1. UPF1 knockdown also resulted in reduced vRNA levels and viral gene expression in HIV-1-infected primary CD4+ T cells.

**Conclusion:**

Overall, these data suggest that RNA surveillance proteins affect HIV-1 gene expression at a post-transcriptional level. An elucidation of the role of vRNA metabolism on the maintenance of HIV-1 persistence can lead to the development of novel curative strategies.

**Electronic supplementary material:**

The online version of this article (10.1186/s12977-018-0425-2) contains supplementary material, which is available to authorized users.

## Background

The implementation of combination antiretroviral therapy (cART) to treat human immunodeficiency virus type 1 (HIV-1) has led the infection to be likened to a chronic condition, with patients on cART having near-normal life expectancy [1]. However, this therapy is not without its drawbacks, such as adverse side effects that lower the adherence rates, the development of drug resistance and its economic repercussions [2–4]. But one of the biggest disadvantages of this therapy is that it is not curative and an infected individual needs to be on cART for the entire duration of their lifetime to effectively suppress viremia. The major hurdle towards an HIV-1 cure is the property of virus to form a stable latent reservoir upon infection that is responsible for the rapid rebound of plasma viral loads when cART is discontinued [5]. This reservoir is primarily composed of resting memory CD4+ T cells along with monocytes and macrophages [6] in peripheral blood and other anatomical compartments such as the gut, lymph nodes and central nervous system. Latency in HIV-1 infection is defined as a reversibly non-productive state of infection which is characterised by the presence of infected cells that do not actively produce viral particles, but retain the ability to do so [7]. Latent cells harbour a replication competent proviral DNA integrated in their genomes [8]. Many research groups have studied the functional aspects of the maintenance of latency in cells by investigating the molecular mechanisms leading to a block at the level of transcription (reviewed in [6, 9, 10]). However, certain studies also highlight that co and post-transcriptional events can also contribute to the maintenance of latency in HIV-1 infected cells [11–13]. These include defective splicing of the genomic viral RNA (vRNA) [14], inhibition of nucleocytoplasmic export of vRNA [13, 15, 16] or an impediment to vRNA translation [17, 18]. Thus, in this work, we investigate the role of the RNA surveillance proteins on the post-transcriptional events that are involved in the maintenance of HIV-1 latency.

RNA surveillance is a host quality control mechanism that identifies and degrades unspliced, aberrantly spliced, intron-containing, upstream open reading frame-containing and premature termination codon (PTC)-containing mRNAs to prevent the accumulation of potentially toxic truncated proteins within the cell (reviewed in [19]). A central player in this mechanism is the Up Frameshift Protein 1 (UPF1), an RNA binding protein that has ATPase and RNA helicase activity [20]. It is a multifunctional protein that has defined roles in DNA repair and replication [21, 22], RNA stability [23–25], telomere metabolism [21] and cell cycle progression [22] (reviewed in [26]). Its most characterised function, however, is its role in nonsense-mediated mRNA decay (NMD) during which UPF1 interacts with a family of proteins such as UPF2, UPF3A and UPF3B, a kinase SMG1 and an endonuclease SMG6 resulting in the degradation of aberrant mRNAs (reviewed in [19, 27]). Although NMD was previously implicated only in the degradation of aberrant mRNA, it is now widely accepted that NMD also targets up to 10% of other physiological mRNAs for degradation in response to cellular needs [19, 28–30], including transcripts that contain long 3′UTRs [31].

In order to promote their survival, viruses have evolved numerous strategies to either evade or manipulate the RNA surveillance pathways (reviewed in [32]). Retroviruses, despite containing long 3′UTRs that are recognised by UPF1, are capable of evading NMD by virtue of the presence of RNA stability elements in their genome [33] (reviewed in [34, 35]). In previous studies, our group has demonstrated that HIV-1 not only evades NMD, it also hijacks UPF1 to form an RNP that promotes vRNA stability and nucleocytoplasmic export [36, 37]. This effect may be exerted during the rapid, co-transcriptional association of UPF1 with vRNA during transcription [38]. UPF2, another protein involved in NMD, has been shown to block nucleocytoplasmic export of the vRNA by binding to UPF1 and preventing its nucleocytoplasmic shuttle [37]. Once in the cytoplasm, UPF1 assembles in another distinct RNP on the vRNA resulting in not only the increased stability of the vRNA, but also in its enhanced translation leading to increased levels of the HIV-1 structural protein pr55^Gag^ viral production [36]. Additionally, UPF1 interacts with vRNA in an RNA length-dependent manner and this could contribute to its incorporation into progeny HIV-1 virions [38–41]. Therefore, there is substantial evidence to show that UPF1 can affect vRNA metabolism at different levels.

In this study, we investigated the ability of UPF1 and its associated proteins UPF2 and SMG6 to influence the HIV-1 gene expression and, as a consequence, viral reactivation at a post-transcriptional level by overexpression and siRNA-mediated knockdown studies in cells of the lymphoid lineage. We employed a fluorescence in situ hybridisation/flow cytometry (FISH-Flow) to monitor vRNA expression levels and viral protein production in a latently-infected T cell line. We observed that these proteins can modulate the HIV-1 gene expression and thus the post-transcriptional maintenance of HIV-1 latency. We have also identified the domains responsible for these effects on viral reactivation by mutational studies. Importantly, we also demonstrate a direct effect of UPF1 on vRNA expression in primary HIV-1 infected CD4+ T cells.

## Results

### FISH-Flow can be used to monitor vRNA levels and viral reactivation in J-Lat cells

**Fig. 1:**
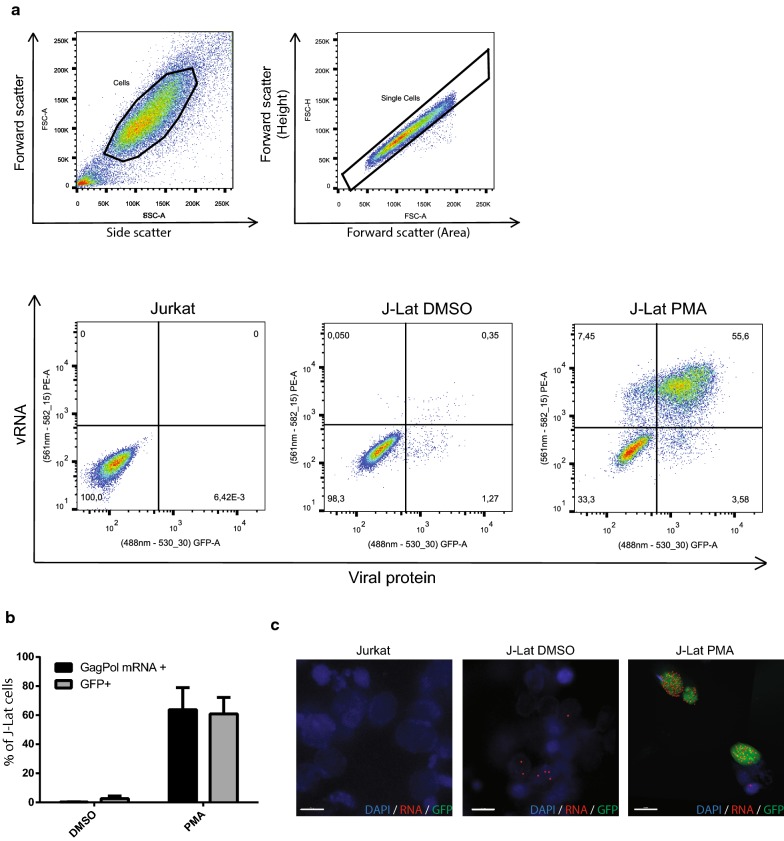
Characterisation of FISH-Flow technique in J-Lat cells. J-Lat cells were either treated with DMSO or with PMA to reactivate the provirus. Jurkat cells were used as an uninfected negative control. **a** Dot plots representing cells gates for size by forward and side scatter, for singlets by forward scatter height versus area and finally for GFP expression and vRNA staining. **b** The % of GFP+ and the % of vRNA-expressing cells were quantified. Error bars represent the standard deviation from three independent experiments. **c** Representative images of cells in each of the above conditions imaged by confocal microscopy. In example images from sorted populations, DAPI is in blue, vRNA in red, and cells making viral protein produce GFP in green. Scale bars represent 10 μm

UPF1 has previously been demonstrated to affect vRNA metabolism at three distinct stages: overall vRNA stability, the nucleocytoplasmic export of the vRNA, and vRNA translation in the cytoplasm [36, 37]. Therefore, we employed the FISH-Flow technique using probes against the GagPol region of the vRNA in latently infected J-Lat 10.6 cells to monitor both the transcriptional as well as translational products of the HIV-1 provirus. This technique has previously been employed to assess ongoing HIV-1 replication, to quantify the size of the inducible latent reservoir in HIV-infected individuals, to determine the kinetics of latency reversal and to characterize the specific cell subpopulations of CD4+ T cells that transcribe HIV-1 RNA [17, 42–44] (reviewed in [45, 46]). Using this technique, it is possible to distinguish between cells that contain both vRNA and viral proteins, and cells that only contain untranslated vRNA, thus differentiating between the transcription-competent and translation-competent viral reservoir [45, 46]. Cells can then also be seeded on a coverslip to determine the sub-cellular localisation of the vRNA using laser scanning confocal microscopy (LCSM). This comprehensive analysis enables us to investigate how UPF1 influences viral reactivation and to distinguish between an effect on vRNA expression, export or translation. J-Lat 10.6 cells, a well-established model of studying HIV-1 latency and reactivation [88, 47, 48], and primary CD4+ T cells are used in this study. The J-Lat cells have a GFP reporter in the *nef* open reading frame of the virus to monitor viral gene expression and, thus, viral reactivation. The cells can be reactivated by treatment with phorbol myristate acetate (PMA) or TNFα (Additional file 1: Figure S1A). To assess whether the FISH-Flow technique can be used in the J-Lat cell model to measure reactivation, cells were either mock treated with dimethyl sulfoxide (DMSO) or treated with PMA to reactivate the cells. PMA is a protein kinase C agonist and is a strong activator of cellular transcription and was the latency reversing agent of choice because it leads to maximal reactivation of the J-Lat 10.6 cells [49]. We also validated the PMA treatment did not affect the baseline expression levels of our proteins of interest: UPF1, UPF2 and SMG6 (Additional file 1: Figure S1B–D). Jurkat cells were used as a negative, uninfected control to determine the specificity of the FISH-Flow technique. Upon treatment with PMA, 60.89 (± 11.35)% of J-Lat cells produced GFP indicating viral protein production and reactivation (Fig. 1a, b). Efficient GagPol mRNA staining was also observed in 63.78 (± 15.16)% of PMA-treated cells. (PE channel, Fig. 1a, b). It is also important to note that 4.79 (± 2.44)% of PMA-treated cells contained vRNA but not GFP, representing the transcription-competent viral reservoir as previously described [45, 46]. The 2.48 (± 1.17) of PMA-treated cells that were GFP+ but did not contain vRNA represent the cells that are generating multiply-transcripts but not full length transcripts, since the GFP codon is present on the *nef* open reading frame [88]. The uninduced J-Lat cells contained some residual vRNA and GFP production, with 2.59 (± 1.76)% of cells expressing GFP and 0.27 (± 0.11)% of cells expressing vRNA (Fig. 1a, b). Although the vRNA is the unspliced genomic viral RNA whereas GFP is generated from the multiply spliced viral RNA, GFP was used as a marker for viral reactivation rather than intracellular p24 due to the efficiency of measuring viral reactivation at a single cell level by Flow cytometry due to the stability of GFP. The levels of pr55^Gag^, coded for by the vRNA, can be measured by Western blot to further correlate effects vRNA transcription and translation, if necessary. Jurkat cells did not show any vRNA+ cells, indicating that this technique is highly specific (Fig. 1a). Cells from each of these conditions were seeded onto coverslips and observed by laser scanning confocal microscopy (Fig. 1c) to view the subcellular localisation of the vRNA. Therefore, the FISH-Flow technique is an efficient method to monitor viral reactivation at the transcriptional and translational levels in J-Lat cells.

### UPF1 knockdown attenuates HIV-1 proviral reactivation

**Fig. 2:**
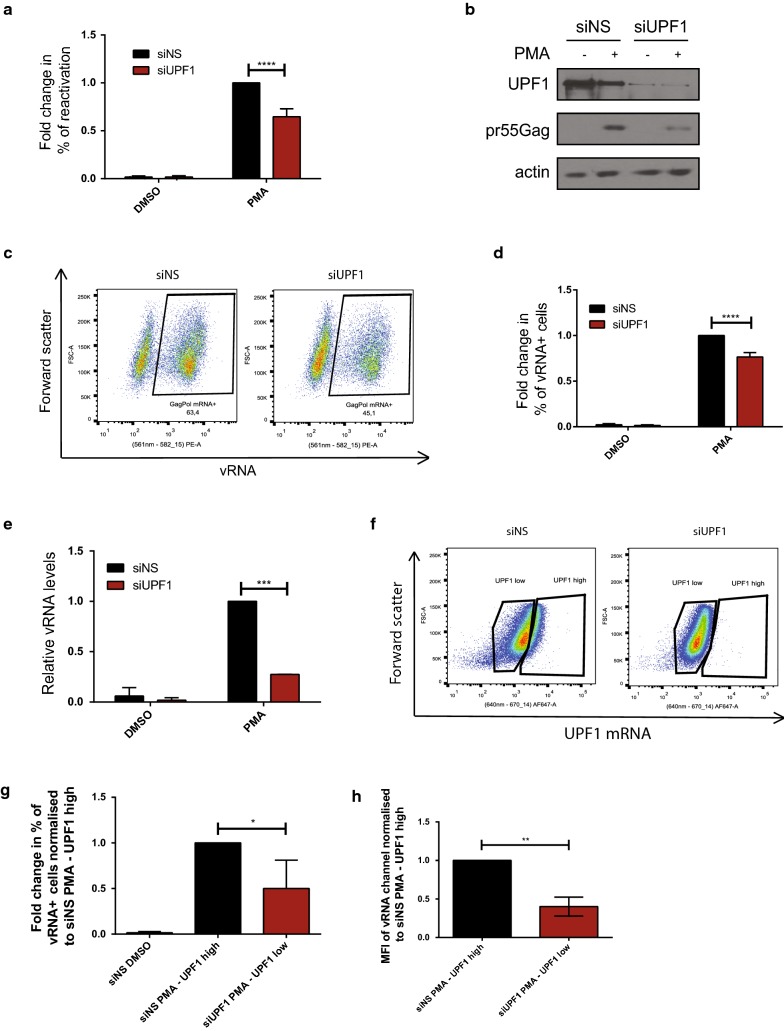
UPF1 knockdown attenuates reactivation of HIV-1 in J-Lat cells. J-Lat 10.6 cells were either transfected with siNS or siUPF1 and were uninduced (DMSO) or reactivated (PMA). **a** Reactivation, monitored by GFP production, was quantified by Flow cytometry and the percentages of reactivation were normalised to the siNS-PMA reactivated condition. Error bars represent the standard deviation from three independent experiments with at least 10,000 cells counted per treatment. Asterisks represent statistically significant difference between groups (Two-way ANOVA; *p* < 0.0001). **b** Cell lysates were run on SDS-PAGE gels and UPF1 and pr55^Gag^ protein levels were detected by Western Blotting. **c** Example dot plot depicting vRNA expression in siNS-PMA and siUPF1 PMA conditions using FISH-Flow technique and, **d** The % of vRNA expressing cells were quantified and normalised to the siNS-PMA condition. Error bars represent the standard deviation from three independent experiments with at least 10,000 cells counted per treatment. Asterisks represent statistically significant difference between groups (Two-way ANOVA; *p* < 0.0001). **e** Levels of vRNA were quantified using RT-qPCR and normalised to the siNS-PMA condition. Error bars represent the standard deviation from two independent experiments, each done in triplicate. Asterisks represent statistically significant difference between groups (Two-way ANOVA; *p* < 0.001). **f** Gating strategy of cells separated into UPF1 low or high by detecting UPF1 mRNA levels by FISH-Flow. **g** The % of vRNA expressing cells in each condition normalised to the siNS-PMA/UPF1-high condition. Error bars represent the standard deviation from three independent experiments. Asterisks represent statistically significant difference between groups (One-way ANOVA; *p* < 0.05). **h** MFI of the vRNA signal were quantified. Asterisks represent statistically significant difference between groups (student’s t-test; *p* < 0.01)

In previous studies conducted by our group, we observed that UPF1 knockdown lead to reduced vRNA stability in the nucleus and in the cytoplasm of cell [36]. Thus, we hypothesised that the depletion of UPF1 can reduce vRNA expression at a post-transcriptional level and thereby inhibit viral reactivation. To evaluate the effect of UPF1 levels on proviral reactivation, J-Lat cells were either transfected with a non-silencing siRNA (siNS) or with siRNA against UPF1 (siUPF1). In each of these conditions, cells were either left uninduced (DMSO) or treated with PMA to reactivate the cells. The percentage of reactivation in the form of GFP production was monitored by flow cytometry and the cell lysates were subjected to Western blotting to validate UPF1 knockdown using antibodies against UPF1, pr55^Gag^ and actin. Treatment of cells with siUPF1 resulted in a 68.9 (± 29.9)% decrease in UPF1 protein levels as measured by Western blot, demonstrating the efficiency of siUPF1 treatment (Additional file 1: Figure S2A). UPF1 knockdown had no significant effect on viral reactivation in the uninduced condition (Fig. 2a). However, upon reactivation with PMA, UPF1 knockdown lead to a 35.3 (± 8.4)% decrease in viral reactivation as compared to the siNS condition (Fig. 2a), which correlated with reduced pr55^Gag^ levels observed by Western blots (Fig. 2b). In order to determine if this decrease in viral reactivation was due to an effect on the vRNA levels or due to inefficient nucleocytoplasmic export or translation of the vRNA, we also conducted FISH-Flow analyses in each of the above reactions. The levels of vRNA were also quantified by RT-qPCR. Upon treatment with PMA, UPF1 knockdown lead to a 23.5 (± 4.8)% decrease in the number of vRNA expressing cells as compared to the siNS treated cells (Fig. 2c, d) as well as a 72.6 (± 0.1)% decrease in the levels of vRNA as quantified by RT-qPCR (Fig. 2e). Of these vRNA expressing cells, a knockdown of UPF1 also led to a 28.0 (± 11.8)% decrease in per cell vRNA levels as measured median fluorescence intensity (MFI) of the vRNA channel (PE) as compared the vRNA in the siNS treated cells (Additional file 1: Figure S2B). This is in accordance with our previous work where we demonstrated that a knockdown of UPF1 resulted in a decrease in vRNA stability [36]. The reduction in vRNA levels as quantified by RT-qPCR in the siUPF1 condition is more dramatic than the reduction of GFP production in the same condition, possibly due to increased stability of GFP as compared to the vRNA. It is also important to note that these detrimental effects of UPF1 knockdown on vRNA levels is specific to the vRNA, since no significant differences were observed in the % of cell expressing a housekeeping mRNA RPL13A and the MFI of the RPL13A mRNA channel measured by FISH-Flow, or in the relative levels of housekeeping mRNA GAPDH measured by RT-qPCR (Additional file 1: Figure S2C–E). However, in these experimental conditions, we can not differentiate between cells that have successful knockdown of UPF1 and non-transfected cells. Therefore, to partially overcome this caveat, we also stained the cells with a UPF1 mRNA probe and, using FISH-Flow analysis, we delineated between UPF1 high vs. UPF1 low cells (Fig. 2f). Using this gating strategy, it was observed that the UPF1 low population of the siUPF1-PMA treated cells showed a 50.5 (± 31.07) reduction in the % of vRNA-expressing cells as compared to the UPF1 high population of the siNS-PMA condition (Fig. 2g). Of these vRNA expressing cells, a knockdown of UPF1 also led to a 1.66 fold reduction in the median fluorescence intensity (MFI) of the vRNA channel (PE) as compared the vRNA in the siNS treated cells (Fig. 2h). Since UPF1 has previously characterised roles in nuclear export [37], we determined if a knockdown of UPF1 resulted in increased nuclear retention of the vRNA. Cellular fractionation was performed and the vRNA present in whole cell, cytoplasmic and nuclear fractions were quantified by RT-PCR (Additional file 1: Figure S3A, B). A decrease is vRNA levels was observed in all fractions, thus implying that in these experimental conditions, UPF1 is acting on vRNA expression rather than on nuclear export (Additional file 1: Figure S3A, B). Taken together, these data suggest that a knockdown of UPF1 leads to attenuated HIV-1 proviral reactivation in J-Lat cells at a post-transcriptional level, by reducing vRNA levels and thus, viral reactivation and protein production.

### UPF1 overexpression enhances HIV-1 proviral reactivation by enhancing vRNA levels

**Fig. 3:**
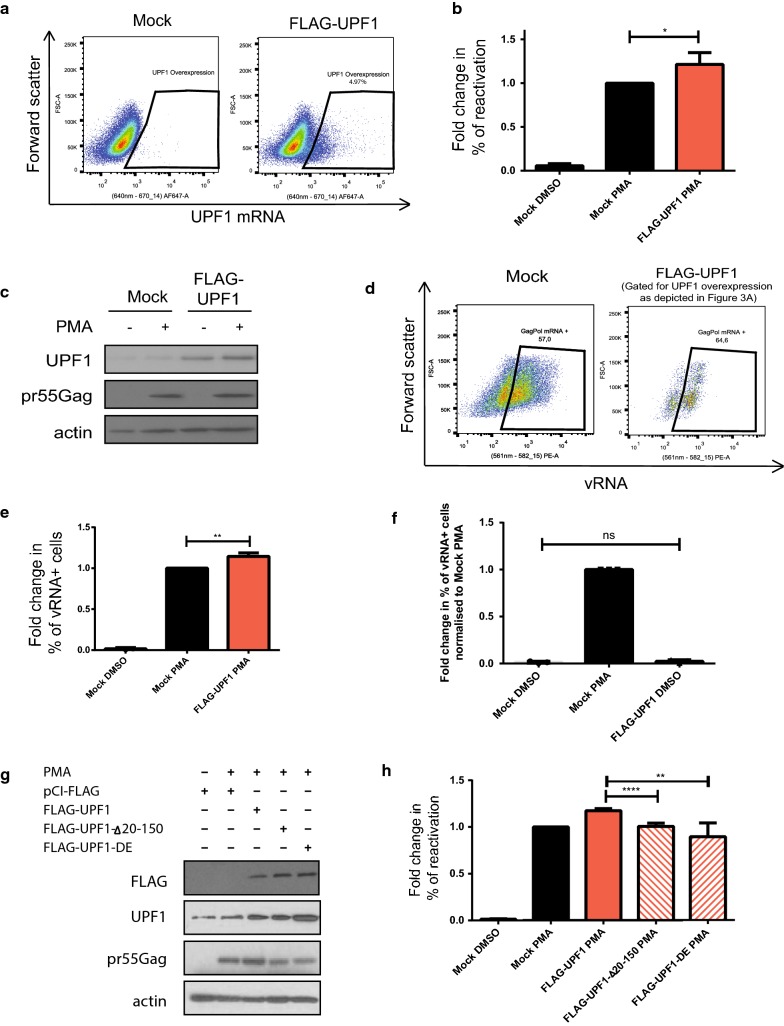
UPF1 overexpression leads to enhanced reactivation of HIV-1 in J-Lat cells. J-Lat 10.6 cells were either mock transfected or transfected with Flag-UPF1 and were uninduced (DMSO) or reactivated (PMA). **a** Gating strategy to detect UPF1 overexpressing cells by detecting UPF1 mRNA levels by FISH-Flow. **b** Of the UPF1 overexpressing cells gated for in **a**, reactivation, monitored by GFP production, was quantified by flow cytometry and the percentages of reactivation were normalised to the mock-PMA reactivated condition. Error bars represent the standard deviation from three independent experiments with at least 10,000 cells counted per treatment. Asterisks represent statistically significant difference between groups (Two-way ANOVA; *p* < 0.05). **c** Cell lysates were run on acrylamide gels and UPF1 and pr55^Gag^ protein levels were detected by Western Blotting. **d** Example dot plot depicting vRNA expression in mock transfected and UPF1 overexpressing populations using FISH-Flow technique. **e** The % of vRNA expressing cells were quantified and normalised to the mock-PMA condition. Error bars represent the standard deviation from three independent experiments. Asterisks represent statistically significant difference between groups (One-way ANOVA; *p* < 0.01). **f** J-Lat cells were either mock transfected and uninduced (Mock DMSO), mock transfected and reactivated with PMA (Mock PMA) or transfected with FLAG-UPF1 and left uninduced (FLAG-UPF1 DMSO). The % of vRNA expressing cells were quantified. Error bars represent the standard deviation from three independent experiments. Asterisks represent statistically significant difference between groups (One-way ANOVA; *p* > 0.05). **g** J-Lat cells were mock transfected or transfected with FLAG-UPF1, FLAG-UPF1-Δ20-150 or FLAG-UPF1-DE and reactivated using PMA. Cell lysates were run on SDS-PAGE gels and UPF1 and pr55^Gag^ protein levels were detected by Western Blotting. **h** Reactivation was quantified in FLAG-UPF1-Δ20-150 and FLAG-UPF1-DE expressing cells and the percentages of reactivation were normalised to the mock-PMA reactivated condition. Error bars represent the standard deviation from three independent experiments with at least 10,000 cells counted per treatment. Asterisks represent statistically significant difference between groups (One-way ANOVA; *p* < 0.0001 and *p* < 0.05 respectively)

UPF1 overexpression has been shown to enhance vRNA stability, nucleocytoplasmic export and translation in previous studies [36, 37]. Therefore, we hypothesised that UPF1 overexpression could enhance proviral reactivation. J-Lat cells were either mock transfected or transfected with FLAG-UPF1. They were then either left uninduced (DMSO) or reactivated with PMA. We employed the FISH-Flow technique using probes against the vRNA as well as UPF1 mRNA to gate for UPF1-overexpressing populations (Fig. 3a). The percentage of reactivation was monitored by flow cytometry and the cell lysates were subjected to Western blotting to validate UPF1 overexpression using antibodies against UPF1, pr55^Gag^ and actin (Fig. 3b, c). UPF1 overexpression resulted in a 21.3 (± 13.5)% increase in viral reactivation upon PMA treatment as compared to the mock-transfected condition (Fig. 3b). UPF1 overexpression also led to a 14.4 (± 4.2)% increase in vRNA levels in the UPF1 overexpressing cells and compared to the mock transfected cells (Fig. 3d, e). UPF1 overexpression in uninduced condition shows no increase in % of vRNA cells as demonstrated by FISH-Flow (Fig. 3f), indicating that UPF1 alone is unable to activate transcription of the provirus and PMA is necessary for transcription to take place. UPF1 overexpression also does not result in a change in the % of vRNA+/GFP-cells as compared to mock treated cells (Additional file 1: Figure S3C). This implies that enhanced viral reactivation upon UPF1 overexpression is due to an effect on vRNA levels rather than an increase in the translation of the transcriptional-competent reservoir. Hence, UPF1 overexpression enhances proviral reactivation at a post-transcriptional level by modestly increasing the expression of the vRNA, thereby resulting in enhanced viral reactivation. This is consistent with our previous work where we demonstrated that an overexpression of UPF1 results in enhanced vRNA stability [36].

In order to determine which domain of UPF1 is responsible for enhancing vRNA expression, we either mock transfected cells, or transfected them with FLAG-UPF1 or other constructs of UPF1 that contain deletions in the N-terminal region (FLAG-UPF1-Δ20-150), deletions in the C-terminal (FLAG-UPF1-1-1074), mutations in the RNA helicase domain of UPF1 (FLAG-UPF1-RR857AA), mutations leading to a deficiency in UPF2 binding ability (FLAG-UPF1-LECY) or mutations in the ATPase region of UPF1 (FLAG-UPF1-DE). These cells were then treated with PMA and the % of reactivation was monitored by flow cytometry (Additional file 1: Figure S4A). The ability of UPF1 overexpression to enhance viral reactivation was lost when the FLAG-UPF1-Δ20-150 construct, which contains an N-terminal deletion or the FLAG-UPF1-DE, that has impaired ATPase activity, were used (Fig. 3g, h). The overexpression of these UPF1 mutants resulted in reactivation at levels comparable to the mock transfected cells treated with PMA. These results indicate that the N-terminal domain and ATPase activity of UPF1 are necessary for its mild effect on enhancing vRNA expression and are consistent with our previous work [36].

### UPF2 overexpression attenuates HIV-1 reactivation via an interaction with UPF1

**Fig. 4:**
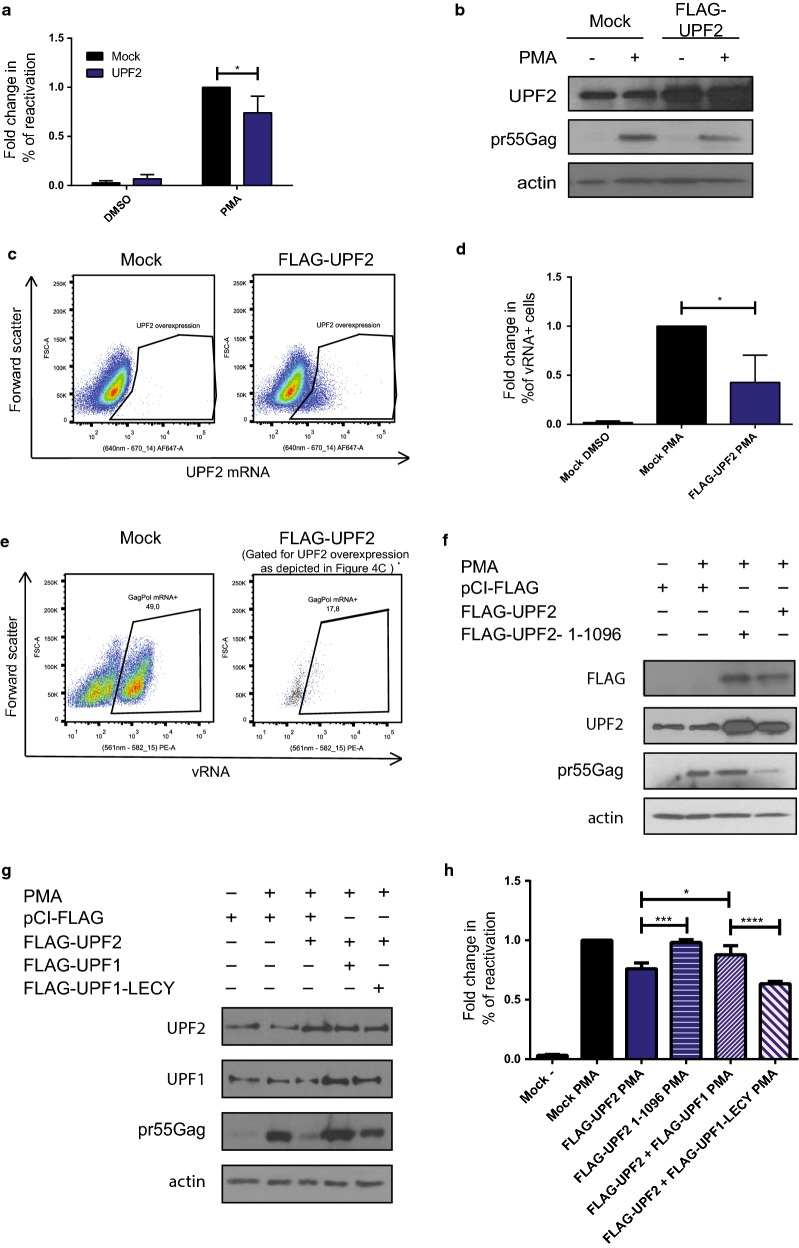
UPF2 overexpression inhibits the reactivation of HIV-1 in J-Lat cells. J-Lat 10.6 cells were either mock transfected or transfected with Flag-UPF2 and were uninduced (DMSO) or reactivated (PMA). **a** Reactivation, monitored by GFP production, was quantified by flow cytometry and the percentages of reactivation were normalised to the mock-PMA reactivated condition. Error bars represent the standard deviation from three independent experiments with at least 10,000 cells counted per treatment. Asterisks represent statistically significant difference between groups (Two-way ANOVA; *p* < 0.05). **b** Cell lysates were run on SDS-PAGE gels and UPF2 and pr55^Gag^ protein levels were detected by Western Blotting. **c** Gating strategy to detect UPF2 overexpressing cells by detecting UPF2 mRNA levels by FISH-Flow. **d** Of the UPF2-mRNA expressing cells gated for in (**c**), the % of vRNA expressing cells were quantified. Error bars represent the standard deviation from three independent experiments. Asterisks represent statistically significant difference between groups (One-way ANOVA; *p* < 0.05). **e** Example dot plot depicting vRNA expression in mock transfected and UPF2 overexpressing populations using FISH-Flow technique. **f** J-Lat cells were mock transfected or transfected with FLAG-UPF2 or FLAG-UPF2-1-1096. Cell lysates were run on acrylamide gels and UPF2 and pr55^Gag^ protein levels were detected by Western Blotting. **g** J-Lat cells were mock transfected, transfected with FLAG-UPF2 or co-transfected with FLAG-UPF1 or FLAG-UPF1-LECY. Cell lysates were run on acrylamide gels and UPF2, UPF1 and pr55^Gag^ protein levels were detected by Western Blotting. **h** Reactivation in the form of GFP expression was quantified in cells transfected as in (**f**) and (**g**) and the percentages of reactivation were normalised to the mock-PMA reactivated condition. Error bars represent the standard deviation from three independent experiments with at least 10,000 cells counted per treatment. Asterisks represent statistically significant difference between groups (One-way ANOVA; *p* < 0.0001)

Previous work from our lab has demonstrated that UPF2 is excluded from the HIV-1 RNP and that its overexpression can block UPF1-mediated nucleocytoplasmic export of vRNA [37]. UPF2 is also known to bind UPF1 with a high affinity [50]. For these reasons, we hypothesised that when UPF2 is present in excess it can sequester UPF1 in the cytoplasm resulting in reduced UPF1 being bound to vRNA. J-Lat cells were either mock transfected or transfected with FLAG-UPF2 and cells were either left uninduced (DMSO) or treated with PMA. The percentage of reactivation in the form of GFP production was monitored by flow cytometry and the cell lysates were subjected to Western blotting to validate UPF2 overexpression using antibodies against UPF2, pr55^Gag^ and actin. Upon reactivation with PMA, UPF2 overexpression resulted in a 25.95 (± 16.8)% decrease in viral reactivation (Fig. 4a) and viral protein production (Fig. 4b). To differentiate between UPF2 overexpressing cells from the whole population and to see if it has any effect on vRNA levels, we conducted FISH-Flow using probes against UPF2 mRNA and vRNA (Fig. 4c). Upon reactivation with PMA, UPF2 overexpression led to a 57.36 (± 27.83) decrease in the percentage of vRNA expressing cells as compared to the mock transfected cells (Fig. 4d, e). Therefore, an overexpression of UPF2 resulted in a modest, albeit statistically significant (*p* < 0.05) decrease in viral reactivation due to a reduction in vRNA expression.

In order to determine if this detrimental effect of UPF2 on vRNA levels is an indirect effect due to its binding to UPF1, we transfected cells with a mutant of UPF2 that does not bind to UPF1 [37, 51, 89] (FLAG-UPF2-1-1096) and compared the % of reactivation in the mock transfected cells, the UPF2 expressing cells and the UPF2-1-1096-expressing cells. It was observed that when UPF2 loses the ability to bind UPF1, there is a loss of its inhibitory effect on reactivation, with reactivation at levels comparable to the mock treated cells (Fig. 4f, h). We also co-transfected FLAG-UPF2 with either FLAG-UPF1 or with FLAG-UPF1-LECY that contains a mutation in the UPF2 binding site and monitored the % of reactivation. UPF1 coexpression is able to rescue the deleterious effect of UPF2 on viral reactivation, but not when in contains a mutation to the UPF2-binding site (Fig. 4g, h). This indicates that the deleterious effect of UPF2 on viral reactivation is a result of its binding to UPF1 which is sequestered and unable to exert a positive effect on vRNA expression, consistent with previous reports [37].

### SMG6 overexpression is detrimental to HIV-1 proviral reactivation

**Fig. 5:**
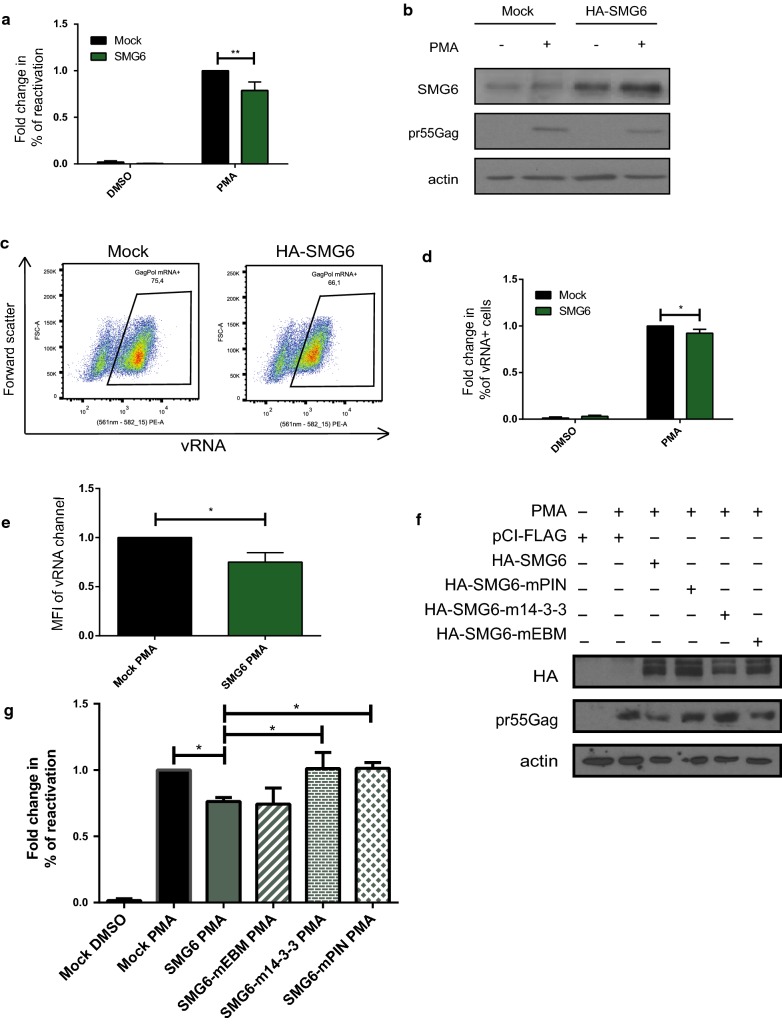
SMG6 overexpression leads to attenuated reactivation of HIV-1. **a** J-Lat 10.6 cells were either mock transfected or transfected with HA-SMG6 and were uninduced (DMSO) or reactivated (PMA). Reactivation, monitored by GFP production, was quantified by flow cytometry and the percentages of reactivation were normalised to the mock-PMA reactivated condition. Error bars represent the standard deviation from three independent experiments with at least 10,000 cells counted per treatment. Asterisks represent statistically significant difference between groups (Two-way ANOVA; *p* < 0.01). **b** Cell lysates were run on acrylamide gels and SMG6 and pr55^Gag^ protein levels were detected by Western Blotting. **c** Example dot plot depicting vRNA expression in mock PMA and SMG6 PMA conditions using FISH-Flow technique. **d** The % of vRNA expressing cells were quantified. Error bars represent the standard deviation from three independent experiments. Asterisks represent statistically significant difference between groups (One-way ANOVA; *p* < 0.05). **e** MFI of the vRNA signal were quantified. Asterisks represent statistically significant difference between groups (student’s t-test; *p* < 0.05). **f** J-Lat cells were mock transfected or transfected with HA-SMG6, HA-SMG6-mEBM, HA-SMG6-m14-3-3 or HA-SMG6-mPIN and reactivated with PMA. Cell lysates were run on acrylamide gels and SMG6 and pr55^Gag^ protein levels were detected by SDS-PAGE followed by Western Blotting. **g** Reactivation in the above conditions was quantified and the percentages of reactivation were normalised to the mock PMA reactivated condition. Error bars represent the standard deviation from three independent experiments with at least 10,000 cells counted per treatment. Asterisks represent statistically significant difference between groups (One-way ANOVA; *p* < 0.05)

UPF1 is an integral member of a network of proteins involved in NMD, including UPF2, UPF3A, UPF3B, SMG6, SMG5, SMG7 and SMG1. SMG6 is the endonuclease involved in the final step of the degradation of aberrant RNA in NMD [52, 53] and has a direct influence on RNA levels. Thus, to evaluate the roles of SMG6 in proviral reactivation, we either mock transfected J-Lat cells or transfected them with HA-SMG6 and either left them uninduced or reactivated them with PMA. The percentage of reactivation in the form of GFP production was monitored by flow cytometry (Fig. 5a) and the cell lysates were subjected to Western blotting to validate SMG6 overexpression using antibodies against SMG6, pr55^Gag^ and actin (Fig. 5b). Overexpression of SMG6 resulted in a 21.2 (± 9.1)% decrease in reactivation (Fig. 5a). Furthermore, upon reactivation with PMA, FISH-Flow analyses revealed a small but significant decrease (7.6 ± 4.1%) in the percentage of vRNA expressing cells upon SMG6 overexpression as compared to the mock-transfected cells (Fig. 5c, d). Of the vRNA present upon SMG6 overexpression, there was a 1.25-fold decrease in the median fluorescence intensity (Fig. 5e). Thus, SMG6 is detrimental to vRNA expression and attenuates PMA-induced proviral reactivation.

SMG6 contains an exon junction binding domain (EBM) [54], a 14-3-3-like domain that binds to phosphorylated UPF1 [55] and a PilT N-terminus (PIN) domain [56] that possesses the endonuclease activity [56–58]. In order to determine which of these domains are responsible for the negative effect on vRNA levels, we transfected J-Lat cells with plasmids that express SMG6 with mutations in each of the aforementioned domains; HA-SMG6-mEBM, HA-SMG6-m14-3-3 and HA-SMG6-mPIN respectively. These cells were reactivated with PMA and the percentage of reactivation was monitored using flow cytometry. While the overexpression of HA-SMG6 and the exon junction binding mutant HA-SMG6-mEBM attenuated proviral reactivation, the overexpression of HA-SMG6-m14-3-3 and HA-SMG6-mPIN displayed reactivation levels similar to the mock transfected cells (Fig. 5f, g). Thus, these results demonstrate that both, the binding of SMG6 to phosphorylated UPF1 and its endonuclease activity are necessary for its inhibitory effect on vRNA levels (Fig. 5f, g).

### SMG6 knockdown increases vRNA expression, but does not affect viral reactivation

**Fig. 6:**
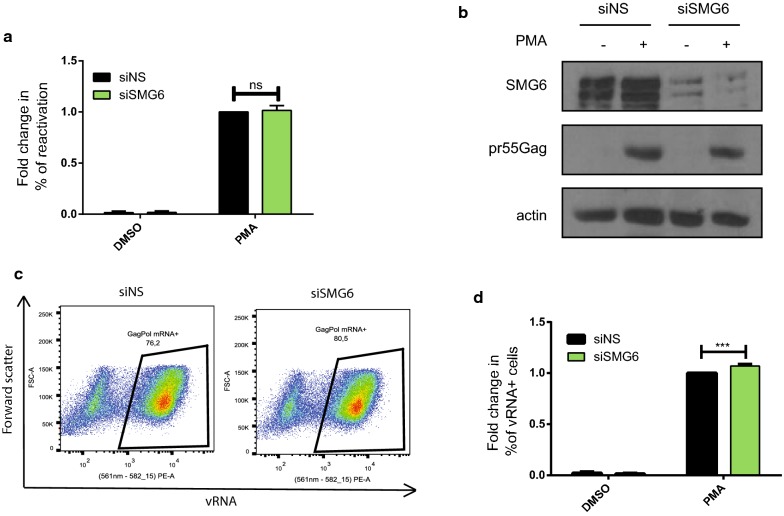
SMG6 knockdown leads to increased vRNA levels, but not reactivation in J-Lat cells. J-Lat 10.6 cells were either transfected either siNS or siSMG6 and were either uninduced (DMSO) or reactivated (PMA). **a** Reactivation monitored by GFP production was measured by flow cytometry. Error bars represent the standard deviation from three independent experiments with at least 10,000 cells counted per treatment. (Two-way ANOVA; *p* > 0.05). **b** Cell lysates were run on acrylamide gels and SMG6 and pr55^Gag^ protein levels were detected by Western Blotting. **c** Example dot plot depicting vRNA expression in siNS PMA and siSMG6 PMA conditions using FISH-Flow technique and, **d** The % of vRNA expressing cells were quantified. Error bars represent the standard deviation from three independent experiments. Asterisks represent statistically significant difference between groups (One-way ANOVA; *p* < 0.001)

To determine the effect of SMG6 depletion on HIV-1 proviral reactivation, we conducted siRNA mediated knockdown studies. J-Lat cells were either transfected with a non-silencing siRNA (siNS) or with siRNA against SMG6 (siSMG6) and cells were either left uninduced (DMSO) or treated with PMA to reactivate the cells. The percentage of reactivation in the form of GFP production was monitored by flow cytometry and the cell lysates were subjected to Western blotting to validate SMG6 knockdown using antibodies against SMG6, pr55^Gag^ and actin. A knockdown of SMG6 did not have a significant effect on viral reactivation at the level of protein production (Fig. 6a, b). However, upon reactivation with PMA and using FISH-Flow using probes against vRNA, SMG6 knockdown resulted in a small but significant increase (6.9 ± 1.8%) in the total number of vRNA expressing cells as compared to the siNS condition (Fig. 6c, d). This further illustrates that SMG6 is detrimental to vRNA expression.

### UPF1 knockdown impairs vRNA expression in primary HIV-1 infected CD4+ T cells

**Fig. 7:**
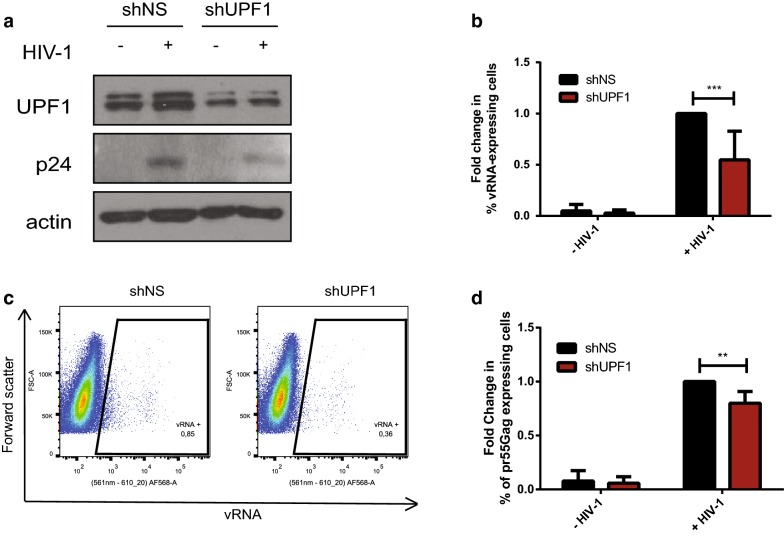
UPF1 knockdown leads to reduced vRNA levels and Gag expression in primary HIV-1 infected CD4+ T cells. Primary CD4+ T cells were either transduced with shNS or shUPF1-containing lentiviral particles and either left uninfected or infected with HIV-1. **a** Cell lysates were run on SDS-PAGE gels and UPF1 and pr55^Gag^ protein levels were detected by Western Blotting. **b** The % of vRNA expressing cells were quantified and normalised to shNS HIV-1-infected condition. Error bars represent the standard deviation from six independent experiments (three donors in duplicate) with at least 5,000,000 cells counted per experiment. Asterisks represent statistically significant difference between groups (Two-way ANOVA; *p* < 0.001). **c** Example dot plot depicting vRNA expression in HIV-1 infected shNS and shUPF1 conditions using FISH-Flow technique and, **d** The % of Gag expressing cells were quantified and normalised to shNS HIV-1-infected condition. Error bars represent the standard deviation from nine independent experiments (three donors in duplicate) with at least 5,000,000 cells counted per experiment. Asterisks represent statistically significant difference between groups (Two-way ANOVA; *p* < 0.01)

UPF1 enhances vRNA expression and, as a consequence, viral reactivation in J-Lat cells. UPF2 and SMG6 are detrimental to vRNA expression, both, via interactions with UPF1. We also assessed the effects of UPF1, UPF2 and SMG6 overexpression on TNFα-induced reactivation of J-Lat cells and observed comparable results (Additional file 1: Figure S4B). However, whether these effects of UPF1 on vRNA expression and pr55^Gag^ expression were also observed in primary CD4+ T cells was yet to be determined. In order to address this question, we conducted shRNA-mediated knockdown of UPF1 in primary CD4+ T cells and observed the effects on vRNA levels and pr55^Gag^ expression upon HIV-1 infection by FISH-Flow. Negatively selected CD4+ T cells from three donors were activated with phytohemagglutinin (PHA). They were then transduced with shUPF1-containing lentiviral particles. Lentiviral particles containing a scrambled sequence were used as a negative control (shNS). The cells were infected with HIV-1 24 h post transduction by spinoculation. Cells were collected 6 days post infection and FISH-Flow was conducted to monitor vRNA and intracellular pr55^Gag^ levels. Cell lysates were also subjected to Western blotting to validate UPF1 knockdown (Fig. 7a). In humans, UPF1 has two isoforms and both isoforms are detected in primary CD4+ T cells [90] (Additional file 1: Figure S5A). However, in J-Lat cells, only the larger one is expressed at high enough levels to be detected by the UPF1 antibody (Additional file 1: Figure S5A). shUPF1 treatment in primary T cells resulted in a 53.8 (± 4.5)% decrease in UPF1 protein levels as compared to the shNS-treated cells (Additional file 1: Figure S5B). Results from three independent donors demonstrated that a knockdown of UPF1 resulted in a 45.16 (± 27.9)% decrease in vRNA levels as compared to the mock treated cells (Fig. 7b, c). This also corresponded with 20.1 (± 10.9)% reduced intracellular pr55^Gag^ staining (Fig. 7d). Therefore, UPF1 also enhances vRNA levels and promotes viral gene expression in primary CD4+ T cells.

## Discussion

The ‘active viral reservoir’ has been defined as the HIV-1 infected cells that contain viral RNA species but do not produce infectious viral particles [59, 60] and this highlights the post-transcriptional maintenance of HIV-1 latency. Latently-infected resting CD4+ cells T cells have been demonstrated to contain cell-associated unspliced and multiply spliced HIV-1 RNA [11, 61]. In these cells, the vRNA was sequestered within the nucleus and could be efficiently rescued through the overexpression of the host protein polypyrimidine tract binding protein (PTB), suggesting that latency can be reversed at a post-transcriptional level [61]. Two characterised primary T cell models of latency have also demonstrated a post-transcriptional block to HIV-1 reactivation, either by sequestration of the vRNA in the nucleus or splicing defects [14, 16, 62]. In addition, microRNAs have been implicated in the maintenance of HIV-1 latency (reviewed in [18]), providing another example of how post-transcriptional events can affect proviral reactivation. In the quest for an HIV-1 cure, the importance of investigating the contribution of post-transcriptional events and vRNA metabolism in the maintenance of HIV-1 latency is being recognised [63–65]. One HIV-1 cure strategy is the ‘shock and kill’ approach which involves reactivating the latent provirus by small molecules (shock) and then to eliminating the virus (kill) using intensive cART and/or immunomodulators [66]. Numerous compounds are under investigation as candidates for latency-reversing agents (LRAs) which promote the transcription of the provirus (reviewed in [67, 68]). So far, the use of LRAs have limited ability to decrease the size of the viral reservoir, with only two reports of successful reduction in reservoir size [7, 69, 70]. The shortcomings of current LRAs is highlighted in a recent study using FISH-Flow in which CD4+ T cells from HIV-1 infected patients were reactivated with the LRAs romidepsin or PMA/ionomycin and only 2–10% of cells that expressed vRNA produced viral proteins [17]. Therefore, the LRAs might be more effective if used in combination with drugs that affect vRNA metabolism at a post-transcriptional level. By modulating the activities of the RNA surveillance proteins or creating small molecules that mimic their activity, we can increase the stability of the vRNA to facilitate reactivation of these latent cells so that they are visible to the immune system and can be targeted by host immune responses and antiretrovirals. Alternatively, we can also apply this study to create novel long-lasting antiretrovirals by designing small molecules to inhibit the binding of UPF1 to vRNA thereby decreasing vRNA stability and reducing viral production.

Using FISH-Flow, this study demonstrates that the RNA surveillance proteins UPF1, SMG6 and UPF2 can affect HIV-1 gene expression, and thus viral reactivation at a post-transcriptional level. Although the effects of UPF1, UPF2 and SMG6 overexpression on modulating viral latency are modest (Figs. 3b, 4a and 5a), these effects nevertheless provide novel evidence of the contribution of post-transcriptional events in viral reactivation from latency. UPF1 was demonstrated to be a positive regulator of viral reactivation in the J-Lat 10.6 latent T cell model. Notably, we also demonstrate a direct effect of UPF1 on enhancing vRNA levels and viral gene expression in primary CD4+ T cells. The overexpression of the ATPase mutant of UPF1 (FLAG-DE-UPF1) did not lead to enhanced reactivation of HIV-1 in J-Lat cells (Fig. 3g, h), indicating that the ATPase activity is responsible for enhanced vRNA expression and viral reactivation. This is in concordance with our previous work where we showed that this UPF1 construct was unable to upregulate vRNA levels and enhance vRNA stability [36]. This ATPase mutant has impaired RNA-binding capacity [71]. To exert its positive effects on vRNA metabolism, UPF1 needs to be able to bind to the vRNA and subsequently lead to the assembly of distinct RNPs that promote vRNA stability, export and translation [37]. An impairment of RNA binding capability could lead to a dissociation of UPF1 from the vRNA, thereby providing another possible explanation why no enhanced viral reactivation was observed when the ATPase mutant of UPF1 was used.

The HIV-1 vRNA metabolism is controlled by numerous cis-acting RNA sequences [72], such as the cis-repressive sequences or instability sequences (INS) [73]. UPF1 contains two zinc fingers that have been implicated to bind to INSs [74] and thus, could promote vRNA stability. The FLAG-UPF1-Δ20-150 construct contains a deletion in the zinc finger motif [36] that could lead to impaired binding to the HIV-1 INS. In agreement with our previous studies where we demonstrate that an overexpression of FLAG-UPF1-Δ20-150 does not lead to enhanced vRNA expression levels [36]; here we demonstrated that, in the context of reversal from viral latency, an overexpression of FLAG-UPF1-Δ20-150 does not lead to enhanced proviral reactivation (Fig. 3g, h), most likely due to impaired binding of UPF1 to the vRNA due to the loss of a zinc finger motif.

We have also previously shown that UPF2 is excluded from HIV-1 RNPs through antagonistic interactions with the viral or host proteins such as Rev or Staufen1 [37]. The binding of UPF2 to UPF1 has been reported to induce a conformational change in UPF1 that stimulates its RNA helicase activity and dampens its RNA binding capability, thereby hampering its binding to the vRNA [75, 76]. UPF2 also binds to UPF1 with high affinity [77] and this could limit the availability of UPF1 to bind to the vRNA. Our data reinforce the hypothesis that UPF2 is detrimental to vRNA metabolism, as we observed that overexpression of UPF2 resulted in reduced vRNA expression and viral reactivation (Fig. 4a–e). This deleterious effect is likely a result UPF2 binding to UPF1 and its sequestration, since viral reactivation was restored to levels similar to control cells when the UPF2 mutant deficient in UPF1 binding was used (Fig. 4f–h). In accordance with our work, a previous report using an shRNA library in J-Lat 5A8 cells showed that shRNAs against UPF1 were disenriched in the reactivated population as compared to the latent population, indicating that it exerts a positive effect on the reactivation of the HIV-1 provirus [78]; whereas shRNAs against UPF2 were enriched in the reactivated population, indicating that UPF2 promotes that maintenance of latency in J-Lat cells [78].

SMG6 is the endonuclease responsible for cleaving mRNAs that are targeted for NMD [52, 53]. Both SMG6 and UPF1 have been reported to be present at transcription sites [79] and SMG6 interacts with UPF1 in a phospho-dependent [55] and a phospho-independent manner [90]. Furthermore, because of its endonuclease activity, SMG6 could have a direct effect on UPF1-bound mRNA levels, such as the vRNA. Our observation that an overexpression of SMG6 results in a decrease of vRNA expression and, consequently, decreased viral reactivation, suggests that SMG6 is detrimental to vRNA stability (Fig. 5a–g). Using mutational studies, we identified that the binding of SMG6 via its 14-3-3 like domain to phosphorylated UPF1 as well its endonuclease activity via its PIN region is necessary to downregulate the viral reactivation (Fig. 5f, g).

Recent transcriptome analyses have demonstrated that UPF1 binds promiscuously to all cellular RNAs; both, canonically identified NMD targets as well as to non-NMD targets and long non-coding RNAs [39, 80–83]. The marker for a cellular NMD target has been revealed to be the RNA’s binding to phosphorylated UPF1 [19, 84]. UPF1 interacts with the PIK-related protein kinase SMG1, SMG8, SMG9, and the two translation termination factors eRF1 and eRF3 to form a decay inducing complex called the SURF [85, 86]. The phosphorylation of UPF1 by SMG1 is necessary for mRNA decay and creates an N-terminal binding platform for SMG6 that cleaves the targeted mRNAs [52, 53, 55]. Hyperphosphorylated UPF1 has been also shown to attract downstream NMD machinery with higher affinity [87]. Therefore, we can speculate that in the context of the interaction between UPF1 and the vRNA, the hyperphosphorylation of UPF1 would be detrimental to vRNA stability due to increased recruitment of SMG6 and other mRNA decay factors. The ATP deficient UPF1 mutant FLAG-UPF1-DE has also been demonstrated to be hyperphosphorylated and assembles complexes with SMG6 on both target and non-target mRNAs [83]. This could provide another possible explanation why the overexpression of the ATPase defective UPF1 did not result in enhanced viral reactivation (Fig. 3g, h). Further investigation is required to elucidate the roles of the phosphorylation status of UPF1 on proviral reactivation.

## Conclusion

In this manuscript, we provide evidence that the RNA surveillance proteins UPF1, UPF2 and SMG6 can affect vRNA expression and thus, the maintenance of HIV-1 latency. These findings can be applied to bolster the reactivation of the HIV-1 provirus to effectively decrease the size of the viral reservoir using a shock and kill approach or can be harnessed to create a novel set of antiretrovirals.

## Methods

### Cell culture

J-Lat 10.6 cells (J-Lat full-length clone 10.6; NIH AIDS Reagent Program) are a Jurkat derived T cell line that is latently infected with HIV-1 in which the *nef* sequence was replaced with a green fluorescent protein (GFP) coding sequence [88]. J-Lat latent proviruses were reactivated by adding 20 ng/mL of phorbol 12-myristate 13-acetate (PMA) (Sigma-Aldrich) to the culture media for 24 h. In case of reactivation with TNFα, 10 ng/ml TNFα (Sigma-Aldrich) was added to the culture media for 24 h. Reactivation of cells was quantified by measuring GFP expression by flow cytometry. All cell cultures were maintained in RPMI 1640 (Life Technologies) supplemented with 10% fetal bovine serum (Hyclone) and 1% penicillin/streptomycin (Life Technologies) at 37 °C and 5% CO_2_. HEK293T cells were purchased from the American Type Culture Collection (ATCC). TZM-bl HeLa cell line was obtained from NIH AIDS Reference and Reagent Program. Both of these cells lines were grown in Dulbecco’s modified Eagle medium (DMEM, Invitrogen) containing 10% fetal bovine serum (HyClone) and 1% penicillin–streptomycin (Invitrogen). PBMCs were isolated from leukophoresed blood collected from healthy donors. All subjects provided informed consent for participating in this study. The research ethics boards of the recruiting sites, the Centre Hospitalier de l’Universite de Montreal and McGill University Health Centre approved this study. PBMCs were isolated by density-gradient centrifugation using lymphocyte separation medium (Corning). CD4+ T cells were negatively selected using the EasySep human T cell enrichment kit according to manufacturer’s protocol (StemCell). Negatively selected CD4+ T cells were maintained in RPMI 1640 (Life Technologies) supplemented with 10% fetal bovine serum (Hyclone) and IL-2 (Sigma-Aldrich). CD4+ T cells were activated by treating them with 10ug/ml PHA (Sigma-Aldrich) for 72 h.

### Antibodies

Mouse anti-p24 was obtained from NIH AIDS Reagents Program; rabbit antisera to UPF1 and UPF2 were generously supplied by Jens Lykke-Andersen (University of California, San Diego, CA, USA); rabbit anti-EST1A (SMG6) and mouse anti-actin were purchased from Abcam; rabbit anti-FLAG was purchased from Sigma-Aldrich; mouse anti-HA was purchased from Roche; mouse anti-GAPDH was purchased from Techni-science; mouse anti-nucleolin was purchased from Santa-Cruz Biochemistry; KC57-FITC was purchased from Beckman Coulter; horseradish peroxidase-conjugated secondary antibodies were purchased from Rockland Immunochemicals.

### Plasmids

The plasmids pCI-FLAG, FLAG-UPF1, FLAG-UPF1-Δ20-150, FLAG-UPF1-1-1074, FLAG-UPF1-RR857AA, FLAG-UPF1-LECY, FLAG-UPF1-DE, FLAG-UPF2 and FLAG-UPF2-1-1096 were described previously [36, 37, 89]. HA-SMG6, HA-SMG6-mEBM, HA-SMG6-m14-3-3 and HA-SMG6-mPIN were a kind gift from Dr. Oliver Muhlemann and are previously described [90]. pNL4.3 was obtained from NIH AIDS Reagents Program.

### Gene silencing

Custom siRNA duplexes were synthesised by Qiagen. The target sequence for UPF1 was 5′-AAGATGCAGTTCCGCTCCATT-3′ and for SMG6 was 5′-GCTGCAGGTTACTTACAAG-3′. The siNS used in this study is a commercially available non-silencing control duplex with target sequence 5′-AATTCTCCGAACGTGTCACGT′-3′.

### Transfections

J-Lat or Jurkat T cells were transfected with either 1 µg of plasmid DNA or 20 nM of siRNA per 1 × 10^6^ cells using the Neon Transfection System (Thermo Fisher Scientific) according to manufacturer’s protocols using the following electroporation parameters: three pulses of 1350 V and 10 ms at a cell density of 1 × 10^7^/mL. J-Lat cells were reactivated 24 h after transfection. HEK293T cells were transfected using JetPrime transfection reagent according to manufacturer’s protocol using 1ul of Jetprime (Polyplus) for 1ug of plasmid DNA.

### Viral transduction

psPAX2, pMD2.G and the pLKO-shNS lentiviral control plasmid containing scrambled non-target shRNA used as a negative control were kind gifts from Dr. Marc Fabian (McGill University). pLKO-shUPF1 (TRCN0000022254) expression vector containing shRNA to UPF1 was obtained from the McGill genetic perturbation service. HEK293T cells were plated in 10 cm-dishes plates and were co-transfected with either shNS or shUPF1 expressing lentivirus, psPAX2 and pMD2.G. Supernatants were collected 48 h post-transfection, passed through a 0.45-μm filter (Pall) and supplemented with 5 μg/ml polybrene (Sigma-Aldrich). The viral particles were added to the primary CD4+ T cells (1 ml of supernatant per 10,000,000 cells) and incubated for 16 h, following which they were infected with HIV-1.

### HIV-1 virus production and infection

NL4.3 virus particles were prepared by transfection of HEK293T cells with HIV-1 NL4-3 provirus-encoding plasmid pNL4.3 using the JetPrime transfection reagent. The supernatants were collected 48 h post transfection, filtered through a 0.45-μm filter (Pall) and centrifuged at 20,000 r.p.m. for 1 h at 4 °C to pellet the virus. Viruses were resuspended in RPMI and stored at − 80 °C. The multiplicity of infection (MOI) of viruses were quantified using the X-gal staining assay in TZM-bl cells as described in [91]. CD4+ T cells in RPMI were infected with an MOI of 0.5 NL4.3 viruses by spinoculation at 1800 r.p.m. for 45 min. Following spinoculation, the cells were washed and replenished with complete culture media. Cells were collected 6 days post infection.

### Western blotting

Cells were lysed in NP40 lysis buffer (50 mM Tris pH 7.4, 150 mM NaCl, 0.5 mM EDTA, 0.5% NP40). Protein concentration on each cell lysate was quantified by Bradford assay. Equal amounts of protein (20 µg) were separated by SDS-PAGE and transferred to a nitrocellulose membrane (Bio-Rad). Blocking was performed using 5% non-fat milk in Tris-buffered saline (pH 7.4) with 0.1% Tween 20 (TBST) for 1 h at room temperature. Membranes were probed with the indicated primary and corresponding horseradish peroxidase-conjugated secondary antibodies. Proteins were detected using Western Lightning Plus-ECL (PerkinElmer). Signal intensities were scanned by densitometry using ImageJ software (NIH, Bethseda, USA).

### FISH-flow

Cells were collected, fixed, permeabilized and subjected to the PrimeFlow RNA assay (Thermo Fisher Scientific) following the manufacturer’s instructions and as described in [42, 92]. For intracellular pr55^Gag^ staining in primary CD4+ T cells, KC57-FITC antibody (Beckman Coulter) was used in permeabilisation buffer from the kit at a dilution of 1:50 for 30 min at room temperature, followed by 30 min at 4 °C. For all samples, mRNA was labelled with a set of 40 probe pairs diluted 1:20 in diluent provided in the kit and hybridized to the target mRNA for 2 h at 40 °C. The probes for GagPol, UPF1, UPF2 and SMG6 used had the following catalog numbers: GagPol HIV-1 VF10-10884, UPF1 VA1-3004200, UPF2 VA1-3007897 and SMG6 VA1-3001031. Positive control probes against the house-keeping gene RPL13A (VA1-13100) were included in each experiment. Samples were washed to remove excess probes and stored overnight in the presence of RNAsin. Signal amplification was then performed by sequential 1.5 h, 40 °C incubations with the pre-amplification and amplification mix. Amplified mRNA was labelled with fluorescently-tagged probes for 1 h at 40 °C. Gates were set on the uninfected Jurkat cells, unstimulated J-Lat control or uninfected primary CD4+ T cells where appropriate. Samples were acquired on a BD LSR Fortessa Analyzer. Analysis was performed using the FlowJo V10 software (Treestar).

### Confocal microscopy following FISH-flow

Cells that underwent the FISH-Flow assay described above were seeded on 18 mm diameter coverslips and air dried. Coverslips were mounted in ProLong Gold Antifade Reagent with DAPI (Life Technologies). Laser scanning confocal microscopy was performed on a Leica DM16000B microscope equipped with a WaveFX spinning disk confocal head (Quorum Technologies) using a 63X objective lens. Images were acquired with a Hamamatsu ImageEM EM-charges coupled device (CCD) camera and image reconstruction was performed with the Imaris software (v. 8.4.1, Bitplane, Inc.).

### RT-qPCR

For data presented in Fig. 2e, total RNA was extracted from cells using Aurum Total RNA Mini kits (Bio-Rad). RT-qPCR analysis of HIV-1 RNA levels was performed as previously described [93, 94]. For data presented in Additional file 1: Figure S2E and Additional file 1: Figure S3B, cellular fractionation was performed as described in [95]. RNA extraction from each fraction were performed using Trizol Reagent (Thermo Fisher Scientific) following manufacturer’s instructions. cDNA was obtained using the High-Capacity cDNA Reverse Transcription Kit (Applied Biosystems). cDNA and primers were then added to GoTaq Green Master Mix (Promega). GAPDH was amplified using the primers GAPDH_1 forward 5′-TGACCACAGTCCATGCCATC-3′ and GAPDH_1 reverse 5′-ATGATGTTCTGGAGAGCCCC-3′ and HIV-1 vRNA using the primers pNL4-3_1 forward 5′-GGGAGCTAGAACGATTCGCA-3′ and pNL4-3_1 reverse 5′-GGATGGTTGTAGCTGTCCCA-3′. The PCR products were visualised in a 1% agarose gel by staining the DNA with RedSafe Nucleic Acid Staining Solution (iNtRON). Signals were captured using a Gel Doc System and intensities were normalised to the GAPDH signal.

### Statistical analysis

All experiments were performed in triplicate, and the data are presented as the mean ± standard deviation (SD). A *p* value of < 0.05 in a student’s t-test, one-way or two-way ANOVA test was considered statistically significant. GraphPad Prism 6 (Graphpad Software Inc.) was used to conduct statistical analyses and create graphs.

## Additional file

**Additional file 1: Figure S1.** **A** J-Lat cells were treated with TNF-alpha or different concentrations of PMA and the % of GFP positive cells were measured. Example dot plot depicting **B** UPF1 mRNA, **C** UPF2 mRNA and **D** SMG6 mRNA expression in mock transfected cells with and without PMA addition using FISH-Flow technique. **Figure S2.** J-Lat 10.6 cells were either transfected with siNS or siUPF1 and were uninduced (DMSO) or reactivated (PMA). **A** Quantification of UPF1 protein expression by densitometry analysis of Western blots. **B** MFI of the vRNA signal were quantified. Asterisks represent statistically significant difference between groups (student’s t-test; *p* < 0.05). **C** The % of RPL13A mRNA expressing cells were quantified. **D** MFI of the PRL13A signal were quantified. **E** Relative GAPDH mRNA levels as measured by RT-PCR. For all graphs, error bars represent the standard deviation from three independent experiments. **Figure S3.** **A** Cellular fractionation was performed in siNS or siUPF1 treated conditions, with and without PMA treatment. The fractions were run on SDS-PAGE gels and GAPDH and nucleolin protein levels were detected by Western Blotting to confirm fractionation. **B** The relative amounts of vRNA in each fraction were quantified by RT-PCR and normalised to levels of GAPDH mRNA. Error bars represent the standard deviation from three independent experiments. **C** J-Lat cells were mock transfected, transfected with FLAG-UPF1, FLAG-UPF2 or HA-SMG6 and reactivated with PMA. The % of vRNA+/GFP-cells was quantified. Error bars represent the standard deviation from three independent experiments. **Figure S4. A** J-Lat cells were mock transfected, transfected with FLAG-UPF1 or with FLAG-UPF1 mutants and reactivated with PMA. Reactivation in the above conditions was quantified and the percentages of reactivation were normalised to the Mock PMA reactivated condition. Error bars represent the standard deviation from three independent experiments with at least 10000 cells counted per treatment. Asterisks represent statistically significant difference between groups. **B** J-Lat cells were mock transfected, transfected with FLAG-UPF1, FLAG-UPF2 or HA-SMG6 and reactivated with TNF-alpha. Reactivation in the above conditions was quantified and the percentages of reactivation were normalised to the Mock PMA reactivated condition. Error bars represent the standard deviation from three independent experiments with at least 10000 cells counted per treatment. **Figure S5. A** Equal amounts of cell lysates from J-Lat 10.6 and primary CD4+ T cells were subjected to Western blotting and probed for UPF1 and actin. **B** Primary CD4+ T cells were either transduced with shNS or shUPF1-containing lentiviral particles and either left uninfected or infected with HIV-1. Quantification of UPF1 protein expression by densitometry analysis of Western blots.
