# Supplementary material for: The RNA surveillance proteins UPF1, UPF2 and SMG6 affect HIV-1 reactivation at a post-transcriptional level
Source: Retrovirology. 2018 Jun 28;15:42. doi: 10.1186/s12977-018-0425-2 (PMC6022449; doi:10.1186/s12977-018-0425-2)

Additional Figure 1 Legends:

Figure S1: A) J-Lat cells were treated with TNF-alpha or different concentrations of PMA and the % of GFP positive cells were measured. Example dot plot depicting B) UPF1 mRNA, C) UPF2 mRNA and D) SMG6 mRNA expression in mock transfected cells with and without PMA addition using FISH-Flow technique.

Figure S2: J-Lat 10.6 cells were either transfected with siNS or siUPF1 and were uninduced (DMSO) or reactivated (PMA). A) Quantification of UPF1 protein expression by densitometry analysis of Western blots. B) MFI of the vRNA signal were quantified. Asterisks represent statistically significant difference between groups (student's t-test; $p < 0.05$). C) The % of RPL13A mRNA expressing cells were quantified. D) MFI of the PRL13A signal were quantified. E) Relative GAPDH mRNA levels as measured by RT-PCR. For all graphs, error bars represent the standard deviation from three independent experiments.

Figure S3: A) Cellular fractionation was performed in siNS or siUPF1 treated conditions, with and without PMA treatment. The fractions were run on SDS-PAGE gels and GAPDH and nucleolin protein levels were detected by Western Blotting to confirm fractionation. B) The relative amounts of vRNA in each fraction were quantified by RT-PCR and normalised to levels of GAPDH mRNA. Error bars represent the standard deviation from three independent experiments. C) J-Lat cells were mock transfected, transfected with FLAG-UPF1, FLAG-UPF2 or HA-SMG6 and reactivated with PMA. The % of vRNA+/GFP- cells was quantified. Error bars represent the standard deviation from three independent experiments.

Figure S4: A) J-Lat cells were mock transfected, transfected with FLAG-UPF1 or with FLAG-UPF1 mutants and reactivated with PMA. Reactivation in the above conditions was quantified and the percentages of reactivation were normalised to the Mock PMA reactivated condition. Error bars represent the standard deviation from three independent experiments with at least 10000 cells counted per treatment. Asterisks represent statistically significant difference between groups. B) J-Lat cells were mock transfected, transfected with FLAG-UPF1, FLAG-UPF2 or HA-SMG6 and reactivated with TNFalpha. Reactivation in the above conditions was quantified and the percentages of reactivation were normalised to the Mock PMA reactivated condition. Error bars represent the standard deviation from three independent experiments with at least 10000 cells counted per treatment.

Figure S5: A) Equal amounts of cell lysates from J-Lat 10.6 and primary CD4+ T cells were subjected to Western blotting and probed for UPF1 and actin. B) Primary CD4+ T cells were either transduced with shNS or shUPF1-containing lentiviral particles and either left uninfected or infected with HIV-1. Quantification of UPF1 protein expression by densitometry analysis of Western blots.

Figure S1

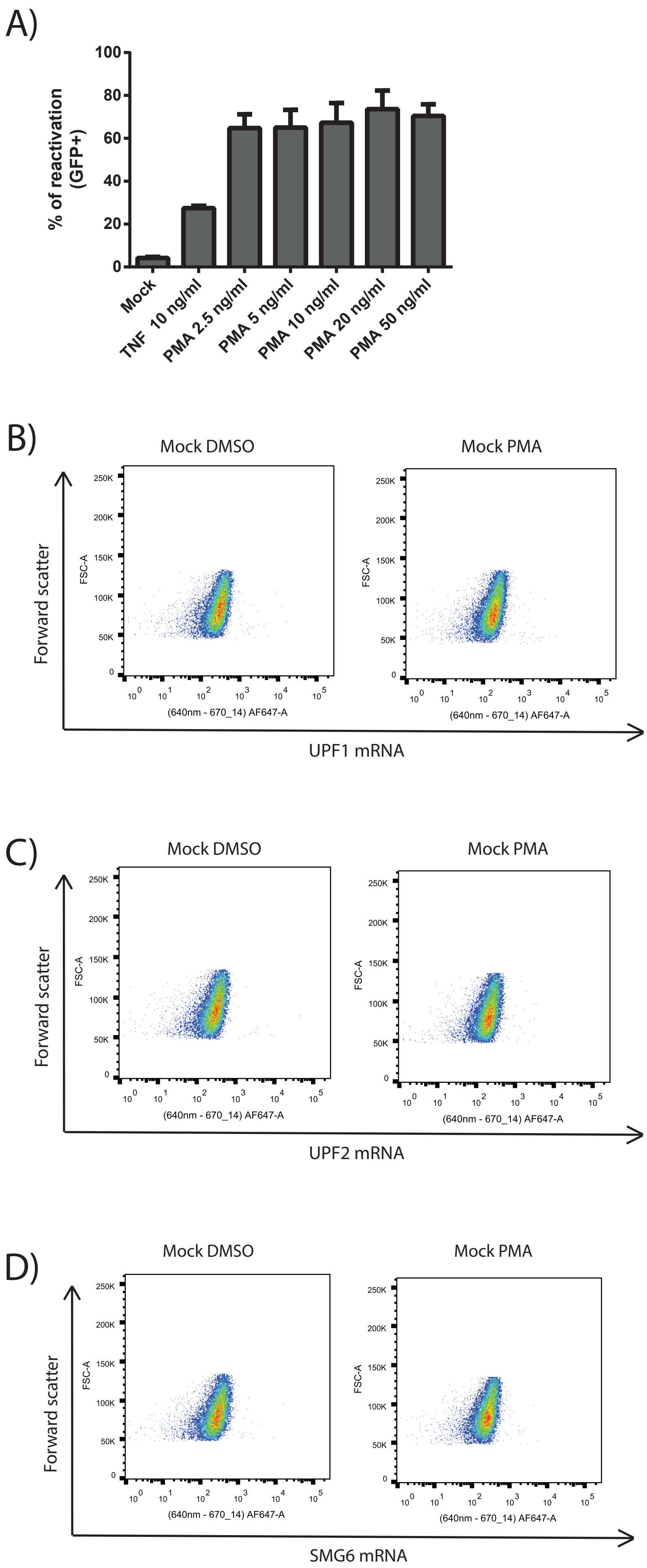
A)
% of reactivation (GFP+)
100
80
60
40
20
0
Mock
TNF 10 ng/ml
PMA 2.5 ng/ml
PMA 5 ng/ml
PMA 10 ng/ml
PMA 20 ng/ml
PMA 50 ng/ml
B)
Mock DMSO
Mock PMA
Forward scatter
FSC-A
(640nm - 670_14) AF647-A
UPF1 mRNA
C)
Mock DMSO
Mock PMA
Forward scatter
FSC-A
(640nm - 670_14) AF647-A
UPF2 mRNA
D)
Mock DMSO
Mock PMA
Forward scatter
FSC-A
(640nm - 670_14) AF647-A
SMG6 mRNA

Figure S2

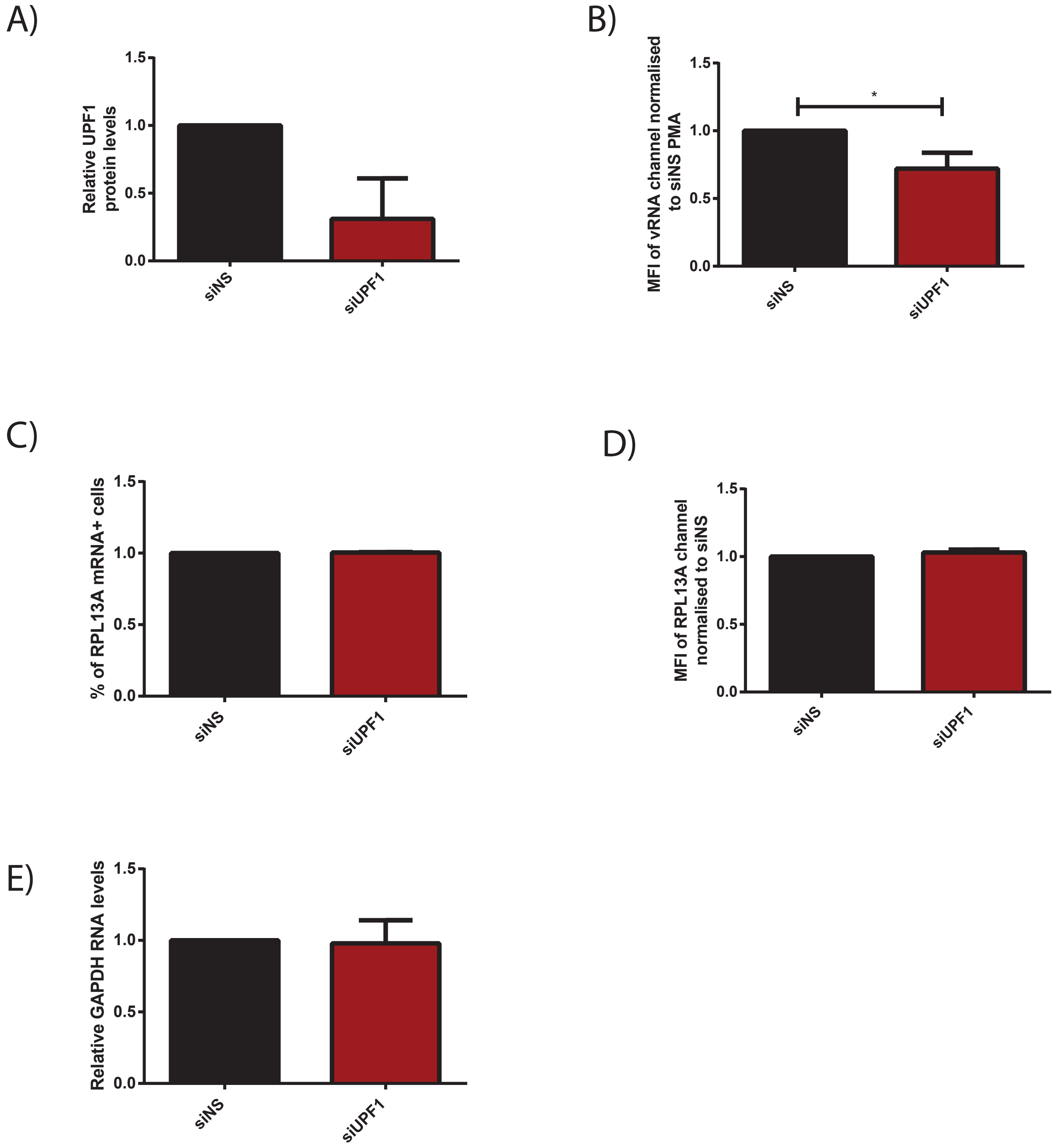
A)
Relative UPF1 protein levels
1.5
1.0
0.5
0.0
siNS
siUPF1
B)
MFI of vRNA channel normalised to siNS PMA
*
1.5
1.0
0.5
0.0
siNS
siUPF1
C)
% of RPL13A mRNA+ cells
1.5
1.0
0.5
0.0
siNS
siUPF1
D)
MFI of RPL13A channel normalised to siNS
1.5
1.0
0.5
0.0
siNS
siUPF1
E)
Relative GAPDH RNA levels
1.5
1.0
0.5
0.0
siNS
siUPF1

Figure S3

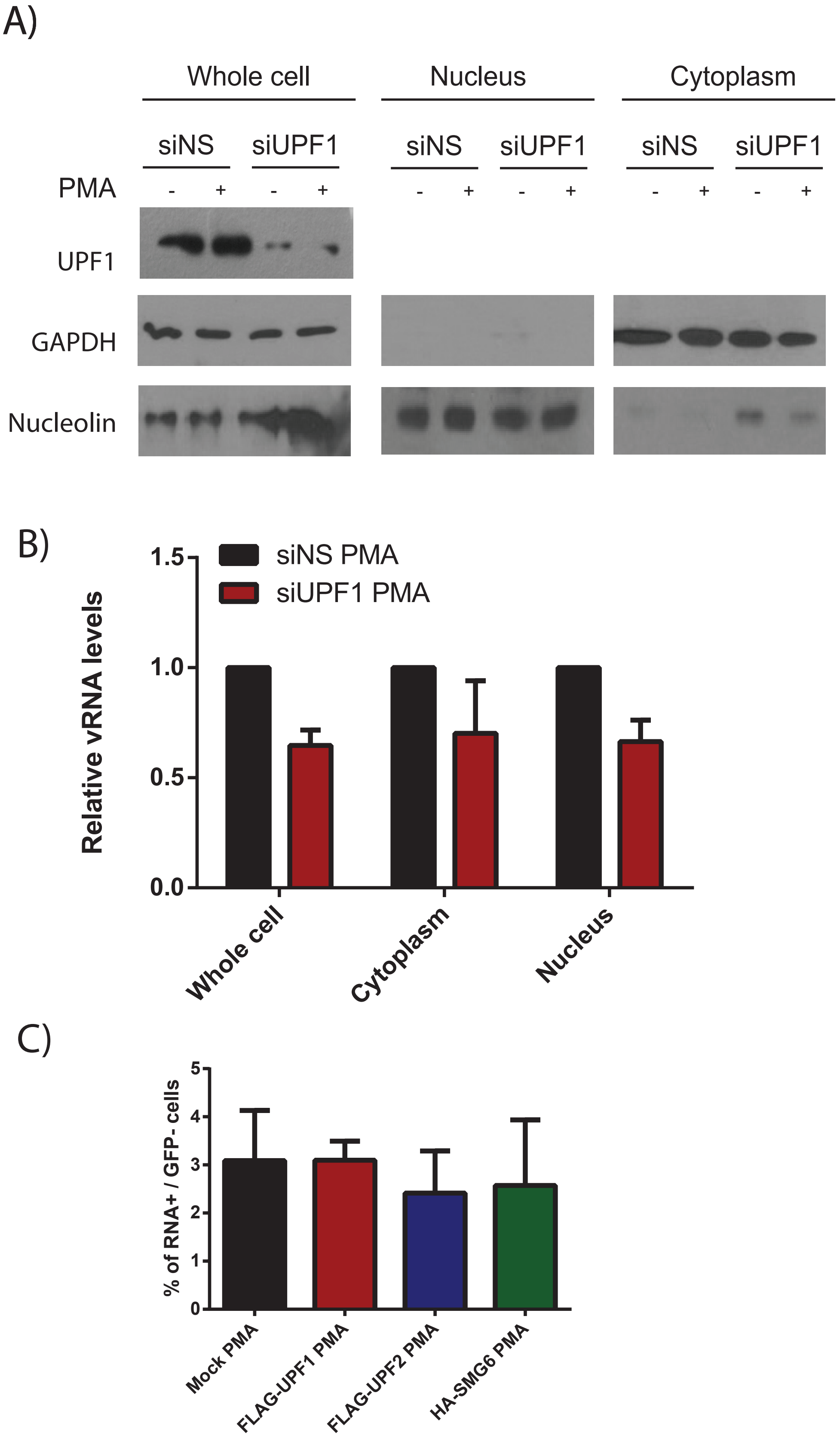
A)
Whole cell
Nucleus
Cytoplasm
siNS
siUPF1
siNS
siUPF1
siNS
siUPF1
PMA
- + - +
- + - +
- + - +
UPF1
GAPDH
Nucleolin
B)
siNS PMA
siUPF1 PMA
Relative vRNA levels
1.5
1.0
0.5
0.0
Whole cell
Cytoplasm
Nucleus
C)
% of RNA+ / GFP- cells
5
4
3
2
1
0
Mock PMA
FLAG-UPF1 PMA
FLAG-UPF2 PMA
HA-SMG6 PMA

Figure S4

A)

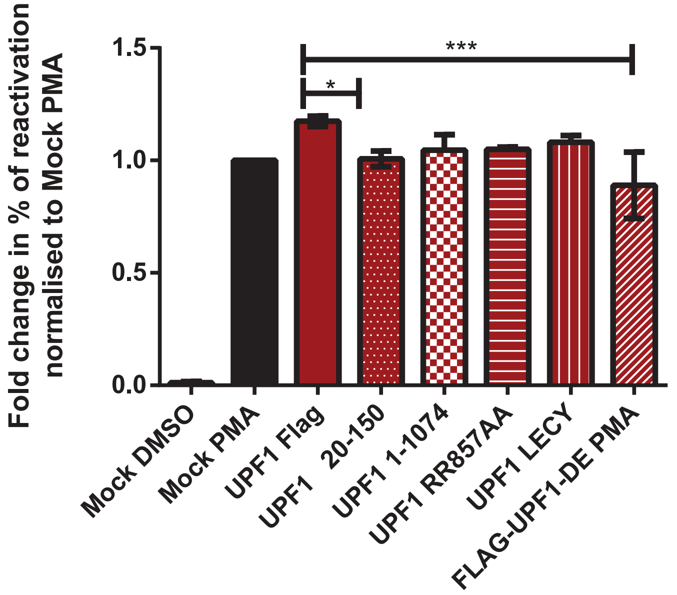

B)

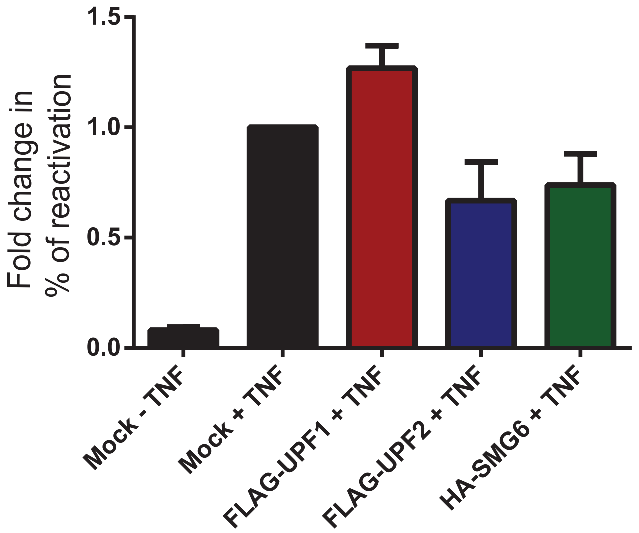

Figure S5

A)

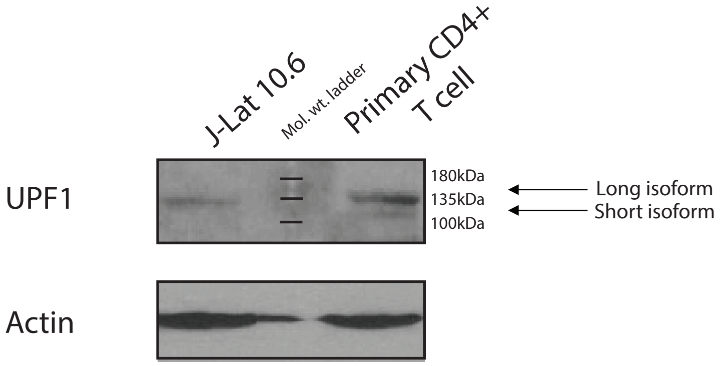

B)

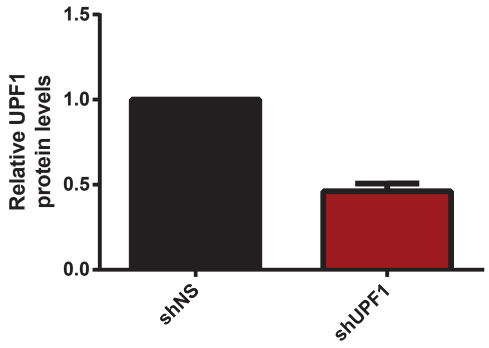

Supplement: Supplementary file 1 — Additional file 1: Figure S1. A J-Lat cells were treated with TNF-alpha or different concentrations of PMA and the % of GFP positive cells were measured. Example dot plot depicting B UPF1 mRNA, C UPF2 mRNA and D SMG6 mRNA expression in mock transfected cells with and without PMA addition using FISH-Flow technique. Figure S2. J-Lat 10.6 cells were either transfected with siNS or siUPF1 and were uninduced (DMSO) or reactivated (PMA). A Quantification of UPF1 protein expression by densitometry analysis of Western blots. B MFI of the vRNA signal were quantified. Asterisks represent statistically significant difference between groups (student’s t-test; p < 0.05). C The % of RPL13A mRNA expressing cells were quantified. D MFI of the PRL13A signal were quantified. E Relative GAPDH mRNA levels as measured by RT-PCR. For all graphs, error bars represent the standard deviation from three independent experiments. Figure S3. A Cellular fractionation was performed in siNS or siUPF1 treated conditions, with and without PMA treatment. The fractions were run on SDS-PAGE gels and GAPDH and nucleolin protein levels were detected by Western Blotting to confirm fractionation. B The relative amounts of vRNA in each fraction were quantified by RT-PCR and normalised to levels of GAPDH mRNA. Error bars represent the standard deviation from three independent experiments. C J-Lat cells were mock transfected, transfected with FLAG-UPF1, FLAG-UPF2 or HA-SMG6 and reactivated with PMA. The % of vRNA+/GFP-cells was quantified. Error bars represent the standard deviation from three independent experiments. Figure S4. A J-Lat cells were mock transfected, transfected with FLAG-UPF1 or with FLAG-UPF1 mutants and reactivated with PMA. Reactivation in the above conditions was quantified and the percentages of reactivation were normalised to the Mock PMA reactivated condition. Error bars represent the standard deviation from three independent experiments with at least 10000 cells counted per treatment. [file 12977_2018_425_MOESM1_ESM.pdf]
